# The Inaugural Dissertation of C. F. J. Bellingeri

**Published:** 1834-10-01

**Authors:** 


### Translation from the Inaugural Dissertation of *Carlo Francisco Joseph Bellingeri*, E. S. Agatha-Derthonensi Philosophiæ et Medicinæ Doctoris, Amplissimi Medicorum Collegii Candidati.

The physiological world has lately been distracted by a controversy on the nervous system. The discoveries which the world has attributed to Sir C. Bell have been snatched at by more than one envious hand, and the wreath has been torn, though it has not withered on his brow. We had hoped to have laid before our readers, upon this occasion, a careful, a candid, and unflinching examination of the claims of some of those who oppose Sir C. Bell. Circumstances delay the publication of the article till the Number of this Journal for January next. A contemporary has advocated, with much warmth and little temper, the right of Bellingeri to be considered the first who elicited and displayed the true functions of the fifth and seventh pair of nerves. With anxious, hurried, and impartial zeal, he has, almost at the same hour, introduced the candidate and chaired the victor. Yet the issue of the triumph *may* remind us of the humorous plate of Hogarth, in which the conqueror's conspicuous situation attracts to himself more weighty bludgeons and more numerous brickbats.

As Signor Bellingeri's claims are hotly urged, the best mode of arriving at a just appreciation of them, is to lay before the public a translation of the essay on which they have been founded. This we have now, through the kindness of a friend, the opportunity of doing. In our next, we shall discuss their nature and their value.

### The Functions of the Fifth and Seventh Pairs of Nerves.

Chapter I.—Art. I. *The Use, Agreement, and Influx of the Major Portion of the Fifth Pair.*

1. Although, in entering upon a much controverted question, I cannot speak with absolute certainty, I shall have enough to adduce upon the ground of fair conjecture. We have, moreover, seen in the previous dissertation, that the fifth pair is distributed to a variety of parts, with entirely distinct uses. Some of these parts are subservient to sense and voluntary motion, some to involuntary or spontaneous action, others, again, to secretion—all, in fact, perform organic functions. With what actions, however, is the fifth pair engaged? Let us consult authority before we decide.

2. Galen has observed that a nerve of the fifth pair, which he designates by the name of the third conjugation, is assigned to the sense of touch and voluntary motion, and that branches of it are distributed throughout the mouth, and subservient to taste.

Willis remarked that the fifth pair attends upon the senses, as those of touch and taste; he maintained, also, that it performed motions, though involuntary or instructive; that it was influenced and excited by sympathy, and by the passions; he also plainly hinted, that the lachrymal branch of the ophthalmic was devoted to involuntary actions (or organic functions), was in a manner subservient to sight and smell, and modified the circulation of the blood in the face.

Vieussens likewise asserted that it was subservient to touch and taste; and that it was also of use in voluntary and involuntary motion, and in the expression of sympathy and passion.

Meckel declared that it expressed the feelings of the mind, and revealed morbid affections in the face; that it directed more of the sympathies of the head, trunk, and limbs; that it presided over the senses of touch and taste, and was even subservient to smell; that it attended upon sight and hearing; and sometimes contracted or dilated the larger bloodvessels and capillaries.

Soëmmering also observed that this pair expressed the sympathies and passions of the mind, and the morbid affections of the viscera. Bichat reckons the nerve of the fifth pair among the nerves of animal life, and transfers the ophthalmic ganglion to the nervous system of organic life. Gall observed that the nerve of the fifth pair is distributed to parts of voluntary motion, and also to parts which are by no means so; that it presides partly over ordinary sensation, as that of touch, partly over special, as that of taste; hence he proposed the term of the mixed pair. Boyer examined the physiology of the fifth pair more fully, for, according to him, it supplies motion to the occipito-frontal and supraciliary muscles, to all the muscles of the face, to the temporal, the pterygoids, the masseter, the muscles of the velum palati, those of the tongue, the mylo-hyoideus, the genio-hyoideus, and to the anterior belly of the digastric; but it supplies sense to the iris, the lachrymal gland, the conjunctiva, the pituitary membrane, the velum and glandular membrane of the palate, the gums, the membrane of the internal mouth, the teeth, the tongue, the tonsillary, maxillary, and sublingual glands, the integuments of the ears, temples, crown of the head, forehead, and the whole of the face.

3. Very numerous, therefore, and diversified are the functions which, in the opinion of these writers, the fifth pair performs. Reason, however, seems to convince us, that this pair eminently contributes to the organic life of the parts to which it is distributed.

4. And in the first place, in an anatomical point of view, its peculiar structure, so admirably adapted to nerves of organic life, the intertextural arrangement of its filaments, its frequent ganglia, the extraordinary number of its anastomoses, the occasionally increased volume of its branches—as we have observed with Boyer, in the trunk of this nerve, in the ciliaries, in the external nasal; and, with the anatomist Scarpa, in the posterior palatine, which constitutes its resemblance to the intercostal; its constant connexion with the arteries, which Bichat considers a distinct characteristic of the organic life of nerves—all these points are in favour of the opinion proposed. Perhaps an argument may also be drawn from its source; for it appears to rise almost entirely from the corpora olivaria, which are properly considered as ganglia.

5. The arguments derived from physiology are still more important. Being actually distributed to the iris, the lachrymal gland, the widely-expanded pituitary membrane of the nose, the maxillary, sphenoidal, and frontal sinuses, the teeth, the internal parts of the ears, all the salivary, the mucous, and the tonsillary glands, the pharynx, and the periosteum, it cannot but perform the functions of organic life. It is true, indeed, that it has reference to the muscles which are subservient to the will and the teguments; but I would have it observed that it is not branches of the fifth pair only which exist here, but that others are immediately added: whilst, for instance, it passes out above and beneath the orbit to the temples, and in the malar region, near the foramen menti, are not filaments of the seventh pair immediately superadded, forming, by a close anastomosis with the branches of the fifth, almost a single nerve? If, then, organic life exists solely where the branches of the fifth pair are exclusively found, as in the lachrymal, nasal, dental, and palatine branches, is it not probable, from its distribution to the muscles and integuments of the forehead, lips, nose, mouth, and entire face, that it attends merely upon (familiari) the organic life of these parts, while their animal life, as voluntary motion and animal sensation, depends on the superadded nerves? Undoubtedly, since the fifth pair supplies the organs of the senses, the organic part depends on this nerve, the animal on those particular nerves which relate to these organs. It is plain that the involuntary motion of the iris, that of the muscles of the internal ear, and the organic life of the pituitary membrane, are regulated by the fifth; yet the sense of smell depends on the first pair, that of sight on the optic, and that of hearing on the acoustic: it seems therefore to follow that both the voluntary motion of the muscles of the face, and the animal sense of touch, depend on the seventh. For, since the branches of the fifth pair exclusively supply the velum palati, with its muscles, and the pharynx, their motion is almost *entirely* removed from the control of the will.

Let us moreover observe, with Gall, that the nerve of the fifth pair is more evolved, in the case of infants newly-born, than any other nerve of the head; and, whereas, in the rest of the animals, a proportion is kept up more than in man, the fifth pair is more remarkable for its thickness. But the life of the fœtus is organic; the life of brutes, also approaches nearer in the face to the organic than that of man. Physiology therefore convinces us that the fifth pair is subservient to organic life.

6. That the fifth pair is principally devoted to organic life is proved, moreover, by actual cases of pathology. It is matter of fact that, in neuralgia, the infra-orbitar branch of the face is divided, and no paralysis of the muscles ensues. The same conclusion is perhaps deducible from the case of the monster described by Lawrence; in which the brain was almost entirely wanting, and the annular protuberance was replaced by a tumor, from which proceeded only the fifth, sixth, eighth, and ninth pairs: the subject lived four days, and took food. Yet it would be fairly inferred that the monster lived an organic rather than a really animal life, from the entire absence of the principal organs which attend upon the latter.

7. Meanwhile, whilst we are proving that the fifth pair attends principally upon organic life, it is also plain that animal life must consequently depend on the same pair: for, upon injury of the organic mode of existence in any part, the animal functions are also of necessity interrupted. Upon this principle we explain the following case:—

Mussano Joseph Antonius, 23 years of age, (an agriculturist of Bibia,) of the sanguine temperament, and of a robust habit, was seized, from seeing an epileptic woman several times, with a severe mental affection, producing disturbed, restless, and terrific sleep; some months afterwards he experienced a heavy pain in the right occipital region, which was followed in a few days by paralysis on the same side of the face. He entered St. John's hospital a month and half after the commencement of the disease, when we observed the following symptoms. There were distortions of the left side of the mouth; pain above and beneath the orbit about the proper foramina, also at the os malæ, and near the foramen menti on the right side; paralysis of the frontal and supraciliary muscles, the orbicularis palpebrarum, the incisor muscles, the canine, the zygomatic, the triangular, and the quadratus menti, and of the orbicularis labiorum on the right side; meanwhile, the motions of the temporal, masseter, and buccinator muscles, and of the tongue, was unimpaired; the deglutition was also undisturbed; the senses of hearing and sight were uninjured; the motion of the iris was perfect in the same part, as also that of the smell in both nostrils, as we proved by experiment. In the mean time, however, there was a diminished sense of titillation in the right nostril, on taking some pungent snuff; and a slight effusion of tears from the right eye; the same kind of snuff being taken in the left nostril, there was greater irritation, and a copious discharge of tears from the left eye; upon stitulating the nostrils with foreign bodies, sternutation ensued on the left side, but none on the right. The sense of taste was considerably impaired and diminished in the right side of the tongue; that of touch was also much obscured in the integuments of the same side of the face. There was also pain in the right scapula. We learn from this case that motion was withdrawn from those muscles, which are supplied by the portio major of the fifth pair, whilst it remained in those which are supplied by the portio minor; that the organic sense of the pituitary membrane was diminished, that its sympathy with the intercostal and lacrymal (nerves) was diminished and almost obliterated; and that the senses of taste and touch were injured, while those of sight, hearing, and smell were unaffected.

7. It is not, however, merely over the organic life of parts that the fifth pair presides, but it also serves to express the involuntary passions and sympathies. The passions of the mind are wonderfully expressed in the face, principally by the branches of the fifth pair. Joy, for instance, is observed in the regular expansion of the frontal muscle, though by a peculiar separation of the muscles of the lips and of the orbicularis oris, by the brightness of the eye, and the natural animated colour of the face, and sometimes, when it is excessive, by a flow of tears. In sorrow, the same branches of nerves are affected, but in an entirely different way; thus the frontal nerves of the fifth, the lacrymal, and the insertions of the infra-orbital in the muscles of the nose and lips express sorrow, and also the branches of the inferior maxillary in the muscles of the chin; the eyes meanwhile droop and are sad, the face is pale and of a dull livid colour.

37. It follows from what has been hitherto said that, with regard to the senses, a distinction is to be made between those of touch and smell, and between organic and animal taste. These senses, so far as they depend on instinct, are referred to organic life; hence they have been denied to no class of animals, and are perfect almost from the period of birth; for it is by these senses that the animals provide for themselves, and preserve their existence; and these senses also act by their connexion with nerves of organic life only, as appears in worms, and in the monster described by Fauvel and by Mery; but animal touch, smell, and taste are performed by distinct nerves, which transmit their sensation to the brain. It is moreover agreeable to the economy of Nature, that the natural or organic senses should be discharged also by the nerves of organic life, but that the animal senses should depend on a distinct class of nerves. The fifth pair, therefore, in our case, presides over the natural or organic senses; but the animal senses, which are found in the same organs to which the fifth is distributed, depend on superadded nerves. I shall therefore decide that the fifth pair is a sentient nerve, and motory of organic life in the head. What I have said generally of the fifth pair, is to be understood only of the portio major of it.

### Art. II. *The Use of the Portio Minor of the Fifth Pair.*

38. The portio minor of the fifth pair has an entirely different origin, course, and structure from the portio major, and is inserted by its proper shoots into the temporal, masseter, and buccinator muscles, the external and internal pterygoids, the orbicularis labiorum, the elevator anguli oris, and the triangularis menti. Palletta asserted that this portio minor performed voluntary motions, and was affected, in trismus, primarily or by sympathy. Since, however, it is to a certain extent subservient, from its insertions, to the taking of food, to deglutition, and especially to mastication, I doubt whether its functions are confined to voluntary motion. Willis observed some time ago—"The taking of food into the mouth is the first and earliest employment of every animal, and is indeed taught by natural instinct prior to all knowledge of the brain;" whence it is plain, that, since the portio minor is engaged in performing these functions, its motions are not always dependant on the will, but sometimes on instinct, as in the infant, who performs all these motions perfectly as soon as he is born. This portion is therefore influenced sometimes by the will, sometimes independently of the will, and by mere instinct. We undoubtedly move all the muscles, which are supplied by the portio minor, voluntarily and at our pleasure. It is plain, however, that this voluntary motion of the muscles depends solely on the portio minor of the fifth, and not on the seventh, or the facial nerve, which is elsewhere united with its branches, from the fact that the portio minor exclusively supplies branches to the fleshy substance of the temporal muscle, and particularly to the pterygoids, without any branches of the facial, and yet that those muscles are under the control of the will; besides, in paralysis of the seventh pair, as will be seen in a case presently to be given, these muscles were actuated by the will. The portio minor therefore relates to the nerves of animal life, and indeed to the motor nerves; for it in no case presides over the senses, and, if its office be considered, it should be termed the nerve of mastication.

Since, therefore, the portio minor is altogether in itself a nerve of animal life, Nature has assigned its origin distinct from that of the major, and, as it is a motor nerve, it arises from the peduncles of the cerebellum. It is therefore evident with what propriety Palletta observed, from anatomical deduction, and from a survey of their different origin, structure, and course, that the crotaphitic and buccinator nerves are distinct from the fifth pair, for their physiological functions also prove that the portio minor is distinct from the major.

39. Having, however, proved that this portio minor performs distinct voluntary motions, we have still to inquire why, in certain cases, it influences the muscles which it supplies, involuntarily or instinctively. This is also shewn by anatomical proof; we have seen, in the preceding dissertation, that the portio minor of the fifth is for the most part intimately connected, at its emergence from the oval foramen, by the gangliform plexus with the inferior maxillary; and, moreover, that almost all the branches of the portio minor receive roots or filaments from the roots of the inferior maxillary; the portio minor is therefore, in its proper ramifications, a nerve composed of its own filaments, and of those of the inferior maxillary: we need not wonder, then, if it perform, generally, voluntary and sometimes involuntary actions, which, however, it effects not by its proper filaments, but by those which proceed from the inferior maxillary, which I think I have already shewn to preside over organic life, and perform involuntary motions. But, besides these latter motions, which the portio minor sometimes produces, it presides in some parts over organic functions, as is proved by the buccinator branch, which is connected by filaments with the duct of Steno, and the buccal glands.

There are then muscles which perform mixed motions, sometimes voluntary, sometimes involuntary; there are also nerves, though these either consist of filaments of different nerves collected into one fasciculus, or the filaments constituting a single nerve have a different origin in the brain.

### Chapter II.—*The Physiology of the Seventh Pair, or the Facial Nerve.*

40. The remarks I am about to make upon the use of the seventh pair, obviously arise from what I have already said, perhaps too fully, respecting the fifth. Let us first explain its physiology; and begin by adducing the words of Rhazes: he has well observed—" It is the fifth pair, (hitherto designated by the single name of the seventh,) by the one part of which occurs the sense of hearing, and by the other are moved the muscles which affect the face." Willis asserted that this nerve performed certain motions, especially those of the passions; that it was affected sympathetically by the acoustic, and expressed the sympathies which occur between the acoustic, the external ear, the eyelids, and the organs of voice—the pharynx, the tongue, and the lips: that it presided over the motions more than over the senses; that it was however subservient to the sense of hearing; he observes, " another (the facial) nerve supplies certain requisites, by which that act (hearing) is more completely performed." Winslow called the facial nerve the small sympathetic. Meckel however affirmed that this nerve was affected by grief; and communicated its proper affection to the diaphragm by the cervical (nerves); that it contracted or relaxed the blood-vessels in affections of the mind; that it performed the sympathy which exists between the lips and the whole of the body in the act of kissing; that it presided over the exquisite sensibility of the lips. Soëmmering said that it expressed laughter in the face, weeping and anger, paleness and redness of the face, and he explained, by means of the facial nerve, the agreement of the teeth with the head, ear, and muscles of the face; and the sympathy of hearing with the eyelids. Gall speaks to the same effect. Bichat reckoned the facial among the nerves of animal life. Boyer said that it imparted motion to the muscles of the stapes, the malleus, and those of the external ear, to the stylo-hyoideus, to the posterior belly of the digastricus, to almost all the muscles of the face, and to the colli-cutaneus. He said that it also presided over sense in the cutis of the head, in the integuments of the ears, of the temples, of the face, and of the anterior and posterior part of the collum.

41. The anatomical structure of the facial nerve suggests to us, that it presides especially over the animal functions in the head, face, and neck, that is, over the animal sense and voluntary motion. It scarcely any where, indeed, forms ganglia; it presents a similar structure to that of the other nerves of animal life, merely constituting remarkable plexuses and frequent anastomoses; it is almost exclusively inserted in the skin and muscles; it should, therefore, be called the cutaneo-muscular of the head and neck. An argument may also, perhaps, be derived from its origin; we see it, in fact, arising principally from the medullary fasciæ, from the corpora restiforma, and from some filaments proceeding from the corpora olivaria. Since then, it chiefly arises from processes of the cerebrum and cerebellum, we infer that it is subservient to sense and animal motion.

42. Physiology also teaches, that the facial nerve is especially devoted to the animal functions. Since, indeed, it supplies the cutis of the head, the face, and the neck, it is rendered very probable, from what we have said on the fifth, that it expresses animal sense; but that, being distributed to all the muscles of the external head and external ears—to almost all those of the face, excepting the temporal, to the stylo-hyoideus, the posteriori digastricus, and colli-cutaneus, it performs animal motion. It should, therefore, be termed from its office, the sentient and motor animal nerve. Its function in the internus of the ears and the parotid we shall examine hereafter.

43. The following is a case of the pathology of the seventh pair.—A patient was lying at St. John's Hospital, under the care of Professor Geri, having been affected for a long time with an inflammatory tumor behind the right ear, which had extended both above and below the mastoid process, so as to compress the facial nerve, at its point of exit from the stylo-mastoid foramen; such was the decided opinion of the Professor, and of Drs. Gallo and Riberi. Meantime, the patient presented almost entire paralysis of the muscles of the right side of the face, and distortion of the left side of the mouth. There was, in fact, complete paralysis of the frontal muscle, the supraciliary, the orbicularis palpebrarum, the elevator alæ nasi, and labii superioris, the caninies zygomaticus, the right side of the orbicularis labiorum, the triangularis and quadratus menti, and colli-cutaneus. The motion of the temporal, masseter, buccinator, and pterygoid muscles, was perfect; of the digastricus we could form no opinion. The motion of the ball of the eye and of the upper eyelid was unimpeded; the vision of the right eye was, however, a little injured; the tongue, also, was moved with some difficulty, yet was the taste proved to be unaffected on either side of the tongue; the sense of touch was also uninjured in the face, the hearing was considerably impaired in the right ear, but the abscess had opened in the external ear. The patient died in about two months. An effusion of pus was found in the cavity of the tympanum, contained in the aqueduct of Fallopius, and compressing the facial nerve in its course; there was no pus or trace of inflammation about the stylo-mastoid foramen after death; but marks of recent inflammation and suppuration in the right lobe of the cerebellum; the fibres and trunk of the fifth pair were uninjured.

As often as the facial nerve is affected with neuralgia, there almost always occurs a spasmodic and convulsive affection of the muscles of the face and neck, as will appear from the following dissertation.

44. The results, also, of *anatomy* shew much more distinctly the physiological use of the seventh pair. Cuvier observes that the facial nerve is invariably small in birds, from their want of lips, a muscular mouth, and almost a face; since there is no sensation and little mobility, there is consequently a very small nerve. On the other hand, the nerve of the fifth pair is observed to be fully developed in birds. I have myself remarked that, in the rabbit, the branches of the facial nerve are very small, but that the branches of the infra-orbital of the fifth are larger and conspicuous; yet, in such creatures, few animal, though many organic, functions are performed in the face.

45. We now proved, by *experiment* on the rabbit, that the seventh pair is subservient to sense and animal motion. We divided the seventh pair, in its course near the parotid, which was followed by paralysis of the muscles of the eyelids, of the upper and lower lip on the same side. On dividing the seventh pair on both sides, nearly in the same place, there was entire paralysis of the eyelids, and of the upper and lower lip; the rabbit still moved the external ears, drew down the lower jaw, and protruded the tongue; the sense of touch appeared to be considerably affected in the face, for, when its hair was plucked, it gave no signs of sensation.

46. Since, then, the seventh pair is subservient to animal life, it produces animal sense in the face and voluntary motion. Since, moreover, it supplies animal sense in the face, branches of the seventh are distributed from this source, together with branches of the fifth, in every part of the head, face, and neck, throughout the skin; and they exist in greater abundance, or more superficially, where the animal touch is more exquisite; thus the seventh forms frequent anastomoses with the subcutanei malarum of the fifth; the shoots of the facial are conspicuous at the lips, and exist in great numbers all over the skin of the neck, imparting to it an exquisite sense, from which the excitement is most easily perceived in these parts. And we have already observed that it presides over the sense of taste, by the chorda tympani, or its lingual branch.

47. But, in the first place, the seventh pair performs almost all the voluntary motions in the face; it moves the muscles of the forehead, the orbicularis palpebrarum; some muscles of the nose evidently, others obscurely, in man, but all of them manifestly in some species of animals, as in the rabbit; also the muscles of the labia, the buccinator, and masseter; all the muscles of the external ear in most animals, if not in man; the occipital, and, at length, the stylo-hyoideus, the mylo-hyoideus, the posterior belly of the digastricus, and the colli-cutaneus, all of which are under the control of the will. But through its lingual branch, it contributes not merely to the sense of taste, but also to the voluntary motion of the tongue, as is proved by the case already given, in which we observed that the voluntary motion was in some degree impeded.

48. Nor is its function confined to the senses and voluntary motions, but it also performs the animal sympathies. By this term, I understand those sympathies which depend on animal sensitiveness and contractility, through the interference of the brain, which are performed with consciousness, and may be prevented by reluctance. Now there are many sympathies which occur between the sense of hearing, the external ear, the eyelids, the tongue, the lips, the larynx, the head, and limbs, through the facial nerve, and its anastomoses with other nerves. Those sympathies, indeed, between the acoustic and facial nerves would be better explained, if the shoot, described by Meckel and Bertinus were a constant one, which enters the interior parts of the ear, and seems to form a part of the acoustic. Moreover, the sympathy between the sense of hearing and the external ear is manifest in the brutes which move their ears voluntarily; for, upon any unusual sound, they erect their ears, and put them, as it were, upon the watch. In man, also, the sympathy of hearing with the eyelids is evident, for we open our eyelids at the slightest sound, and prevent their closing; upon the occurrence of any violent or sudden noise, we almost unconsciously close our eyelids, although we are able to keep them open when accustomed to it, or advised to do so. There is sympathy with the internal and external organs of speech, as the larynx, the tongue, the lips; thus, on hearing a loud noise, fearless animals bellow, and utter terrific howls—the timid, on the contrary, retire in silence; while listening to pleasing sounds, even in the very act, we suppress our voice—we even direct the tone of our voice by the sense of hearing. Since, then, such sympathy exists between the voice and the hearing, we see also that the seventh pair forms an anastomosis with the laryngeal of the par vagum. And from such an anastomosis, we understand how the voice also is obedient to the will; for the voice is an action sometimes voluntary, sometimes instinctive; the infant cries as soon as born. How far does the voluntary depend on the branch of the facial nerve—how far from instinct, arising from the laryngeal branch of the pneumo-gastricus? What limit can be set to the agreement of the tongue with the ear? Thus, it is by hearing we learn fitly to apply our words, whilst the deaf person is also dumb: but the sympathy of the ear and tongue is best explained by the chorda tympani, which is principally a branch of the facial, inserted in the lingual. Nor is the sympathy merely between the hearing and the tongue, but also with the lips and muscles of the mouth; these, consequently, act in utterance, in talking, in singing, and we pronounce all the vowels and labials.

There is also a sympathy of the ear with the whole of the head, and the muscles which move the head; hence, when we hear a slight sound, and are listening attentively, we incline the head, and approach towards the sound—from a louder noise we, as it were instinctively, move away and avert the head. Those animal sympathies which are performed by previous sense and subsequent motion, are best explained by the anastomoses of the facial nerve with the accessory of Willis and the cervicals. By the connexion with the cervicals, we also learn, to a certain extent, how it is that the limbs are variously affected by the sense of hearing; hence, upon hearing an unusual noise, both man and animals attempt voluntarily and immediately to escape; or, on perceiving a loud and violent noise, the man is alarmed, panic-struck, and stands still—if a pleasant and agreeable sound, he keeps his limbs at rest. The deaf man also expresses, by the better significancy of gesticulation, what he cannot utter, and supplies the defect of his tongue with his hands, and the whole of the upper part. The seventh pair having also anastomosed with the cervical, the sympathy of the ear with the lower limbs is in some degree manifest; hence, the soldiers' march is directed by sound, and dancers perform their movements. And thus we see the animal sympathy of the limbs and of the whole body with the face; by gentle tickling of the skin, especially at the soles of the feet and in the palm of the hand, laughing is excited. By these unions with the cervical it cherishes its own sympathy and that which it has with the diaphragm, which is consequently moved in various modes of utterance, in talking, and especially in singing.

49. Besides the animal sympathies, it expresses also the feigned passions of the mind, which consist solely in various movements of the muscles of the face, eyes, and head, in the position of the whole body, in changes of voice, and the utterance of speech. Thus it is with anger, love, joy, sorrow, humility, pride, dignity, which we wholly or in part affect, as is frequently done by actors; and it is with this nerve especially that they enact their buffoonery and provoke laughter. For laughter is also produced by this nerve; and, since there is considerable sympathy between the facial and the acoustic, we are induced to laugh principally through hearing—frequently, also, by animal touch, and sometimes necessarily by sight. Laughter, however, is never produced by the senses of smell, taste, and ordinary touch. The mind is always affected in laughter actively or passively; hence, man is almost exclusively gifted with this faculty.

50. Nor are the motions enumerated the only ones produced by the seventh pair, for, since it is subservient to speech and mastication, it consequently actuates the organs both of voice and of deglutition. For, through the stylo-hyoideus branch, and the intervention of the stylo-hyoideus and mylo-hyoideus muscles, it raises the larynx in deglutition and in speech; and, through the digastricus, it depresses the lower jaw in mastication, in singing, and in groaning. Since, moreover, those muscles are also moved involuntarily, as is seen in the deglutition, voice, and yawning of an infant, it follows that filaments, also, of the glosso-pharyngeal are supplied to these muscles, and the glosso-pharyngeal and facial nerves are united together by a remarkable anastomosis, so as to connect whatsoever there is of the voluntary and involuntary in deglutition and speech.

51. We have now, therefore, seen that the seventh pair attends principally on animal actions; if, however, any one should object, that it presides over involuntary motion in the internal ear, let him refer to our remarks on the fifth pair; let him also observe that it there forms a ganglion, as occurs in the ciliary nerves; since, however, it gives some remarkable branches, also, to the parotid, there will always be a difficulty respecting its subserviency to involuntary motion, and, in some instances, to organic life. Besides, it undoubtedly subserves organic life, since it supplies many filaments to the mastoid cellules, to the membrana tympani, to the longer leg of the malleus, about the foramen jugulare, and to the muscles inserted into the Eustachian tube. But I would observe that the seventh pair is not to be considered as a single nerve, but as consisting of two portions, a major, and a minor discovered by Wrisberg, which has a distinct origin, structure, and a course separate from the portio major. I should, therefore, suspect, that a few of the involuntary actions performed by this seventh pair depend on this portio minor; which would perhaps be more evident if its distributions were understood. In fact, from knowing the distribution of the portio minor of the fifth, we have shewn that the actions it performs are different from those of the portio major; for the minor is subservient to the will, but not the major. Moreover, we can conclude to a certain extent, from conjecture, that the portio minor of the facial is subservient to organic functions; for its filaments appear to rise partly from the substance of the pons varolii, partly from the corpora olivaria, near the origin of the glosso-pharyngeal; but we have already given our opinion that the nervous filaments which rise from the corpora olivaria subserve organic life. I therefore consider that, as the portio major of the fifth is subservient to organic, while its portio minor is devoted to animal life,—so, contrariwise, the portio major of the seventh attends on the animal, and the portio minor on organic life: which being admitted, I will also grant that the muscles of the internal ear are moved by filaments of the portio minor of the facial nerve. Since then the origin and structure are different, and the office of the portio minor of the fifth and seventh pairs distinct from the portio major of each, I fairly consider that each portion constitutes a distinct pair of nerves.

52. We therefore conclude that the facial nerve performs almost all the voluntary motions in the head, presides over animal sense in the face and neck, produces laughter, expresses the feigned affections of the mind, and accomplishes the animal sympathies; hence we also understand why its branches are connected together, and with neighbouring nerves, by frequent anastomoses, as seen by anatomy. We conclude that it acts in mastication, deglutition, voice, speech, singing, and, like the fifth, constitutes a part of all the organic senses; it supplies branches to the internal and external ears, defends the eyes by the eyelids, enters the inferior nostrils, enriches the tongue with a remarkable branch, furnishes the sense of animal touch on the skin, and is subservient every where in these parts to the functions of animal life.

And here I will stop, having spoken too much at large with reference to my ability, but briefly when we consider the wonderful work of the Creator; for in the latter point of view, there would be no limit to our remarks.

Nor is the division of the branches of the fifth pair attended by paralysis of the muscles of the face, or by convulsive trismus, as appears from numerous cases, and from the clear and decisive testimony of Thouret. We accordingly observed, in our physiological remarks, that the portio major of the fifth pair was not subservient to voluntary motion; neither is it attended by atrophy of the parts, for the nerves of organic life appear to perform their peculiar functions partly without the influx of the brain, the medulla oblongata, and spinalis. Allowing, however, that the influx of the brain or marrow were necessary, there is still some communication kept up with these parts by the anastomosing filaments of the infra trochlearis nasalis, and the subcutaneus malæ.

30. Should it be made to appear that neuralgia has attacked the seventh pair, the division of the trunk or its branches is, in my opinion, never to be attempted: I say of the trunk, because, as Langenbeck observes, there is danger of injuring the neighbouring carotid; neither the trunk, indeed, nor the branches should be divided, for it would infallibly be followed by paralysis of very many of the muscles of the face, in respect of voluntary motion.

Jackson says that he divided the facial nerve; but in what part, and with what effect, is not stated. Langenbeck asserts that the zygomatic and facial branches of the seventh pair may be safely divided, but without affording any proof of it. I have, however, shewn by experiment on the rabbit, that voluntary motion ceased, and that the sense of touch was evidently injured in the face, on wounding the facial nerve near the parotid; and in the pathological case of the seventh pair, it was followed by paralysis of the muscles of the forehead, of the lips, and of the mouth. For the seventh pair being a nerve of animal life, its direct communication with the brain is as indispensable for the performance of its proper functions, as in cases of the rest.

I would it were in our power to remove this malady more frequently and more effectually, by careful examination of the affection, and by rigorous investigation of the means we have enumerated.

